# Is sperm morphology functionally related to sperm swimming ability? A case study in a wild passerine bird with male hierarchies

**DOI:** 10.1186/s12862-018-1260-8

**Published:** 2018-09-19

**Authors:** Alfonso Rojas Mora, Magali Meniri, Sabrina Ciprietti, Fabrice Helfenstein

**Affiliations:** 1 Laboratory of Evolutionary Ecophysiology, Institute of Biology, Faculty of Sciences, University of Neuchatel, Neuchatel, Switzerland; 2 Present Address: Laboratory of Ecology and Epidemiology of Parasites, Institute of Biology, Faculty of Sciences, University of Neuchatel, Rue Emile-Argand 11, 2000 Neuchatel, Switzerland

**Keywords:** Sperm morphology, Sperm function, Social dominance, House sparrow

## Abstract

**Background:**

Sexual selection continues after copulation via either sperm competition or cryptic female choice, and favors sperm traits that maximize sperm competitiveness. Both sperm swimming velocity and longevity are important determinants of the outcome of sperm competition. Theoretically, sperm morphology can influence sperm velocity at least in three different non-exclusive ways: (i) longer sperm may generate more propelling thrust, (ii) bigger midpieces may produce more energy, and/or (iii) larger flagella or mid-pieces relative to the head size may compensate for the drag forces around the head. A growing number of studies have investigated the relationship of sperm morphology with sperm performance, which remains equivocal at both the inter- and intra-specific levels. Here, we used House Sparrows to test the functional relationship between sperm morphology with sperm velocity and longevity. Based on a previous study showing that sperm swimming ability covaries with social rank, we predicted that —if a functional relationship exists—1) sperm morphology should differ across social ranks, and 2) correlations between sperm morphology and sperm velocity and/or sperm longevity should be constant across social ranks.

**Results:**

We found no differences in sperm morphology across social ranks. Moreover, we found that sperm morphology may be correlated with sperm velocity, but such relationship varied across social ranks. This result contradicts the hypothesis of a functional relationship between sperm morphology and sperm performance. Finally, after experimentally manipulating social ranks, we observed that relationships between sperm morphology and sperm velocity and/or sperm longevity disappeared or changed direction.

**Conclusions:**

We suggest that in species with internal fertilization, while sperm morphology is likely constrained by the morphology of the female sperm storage organs, selection may act upon physiological traits that enhance sperm performance. Hence, these two selection forces could decouple sperm performance from sperm morphology.

**Electronic supplementary material:**

The online version of this article (10.1186/s12862-018-1260-8) contains supplementary material, which is available to authorized users.

## Background

When females copulate with more than one male, strong selection acts upon traits that maximize sperm competitiveness [1–3]. Postcopulatory sexual selection can be the result of either cryptic female choice or competition between sperm of different males, known as sperm competition, and both are hypothesized to drive the evolution of sperm morphology [1, 4, 5]. For instance, in many taxa sperm morphology has coevolved with that of the female reproductive tract reviewed in [6], and in birds it has been observed that sperm storage tubule length correlates with sperm length [7–10]. Moreover, across taxa it has also been observed that sperm morphology correlates with the intensity of sperm competition [11–18]. However, we understand less well how differences in sperm morphological traits translate into better competitive fertilization success [19].

Sperm velocity has been found to be a major determinant of sperm fertilization success in invertebrates [20], fish [21, 22], mammals [23, 24], and birds [25, 26]. Among the various non-exclusive ways in which sperm design can increase sperm velocity, it has been proposed that (1) longer sperm can benefit from a higher flagellar thrust [13], (2) longer midpieces can both produce larger amounts of energy to fuel the flagellar thrust [27] and –in the case of bird sperm- stabilize the flagellum during the screw-like motion [28], (3) and/or longer flagella or midpieces relative to the head size can compensate for the drag forces around the head by providing either more energy (i.e. midpiece) or propelling power (i.e. flagellum) [29]. Evidence that longer sperm have higher swimming velocities exists in various taxa [13, 28, 30, 31]. Similarly, mid-piece length has been found to positively correlate with swimming speed [27, 32]. Sperm morphology has also been hypothesized to affect other traits than sperm velocity. For example, longer mid-pieces relative to the size of the spermatozoon may prevent the energy reserves from being exhausted, thus sustaining protracted viability. In support of this hypothesis, spermatozoa with longer mid-pieces relative to head size have been found to live longer [33].

When access to fertile females is biased towards males of a given phenotype or engaged in different social roles (e.g. dominant vs. subordinate, territorial vs. sneaker), theory predicts that as males have lower access to females they should increase expenditure into post-copulatory traits [3, 34, 35]. Evidence validating the predictions of these models has been found in insects [36], fish [37, 38], birds [39], and mammals [40, 41]. Consequently, if sperm morphology functionally correlates with sperm performance, we predict that in species where male dominance covaries with sperm performance, sperm morphology should also covary with male dominance. However, predictions on the direction of the differences in sperm morphology across a dominance hierarchy are difficult to formulate, given that the functional relation between sperm morphology and function is as yet unclear.

House sparrows (*Passer domesticus*) are socially monogamous passerine birds exhibiting significant levels of sperm competition and extrapair paternity (12–18%) resulting from female promiscuity and forced copulations [42–47]. It is worth noting that although older house sparrow males seem to monopolize extra-pair paternities [43], Møller [48] has shown that more dominant males obtain more extra-pair copulations. Moreover, we have shown that male House Sparrows occupying different social ranks produce sperm performing differently [49]. In the current study, we used data from this previous study [49] and added morphological measurements of spermatozoa to test whether sperm performance is functionally related to sperm morphology. We predicted that, if sperm morphology explains variation in sperm performance, 1) sperm morphology should explain the differences we found in sperm performance across social ranks and should thus differ across social ranks, and 2) the direction of any correlations between sperm morphology and sperm velocity and/or sperm longevity should remain qualitatively the same across all social ranks.

## Results

### Sperm morphology and social status

Before manipulating the social status, we were unable to obtain a sperm sample from one male, thus our results are based on ejaculates from 59 males. We did not find any differences in total sperm length or sperm design in relation to male social rank (Table 1). However, total sperm length was positively correlated with male body mass (Table 1). After manipulating the social status, the newly stablished hierarchical ranks did not explain variation in total sperm length and sperm design (Table 2). However, for both total sperm length and sperm design a significant interaction arose between social rank and body mass (Table 2). Specifically, while for dominant males both total sperm length and sperm design decrease with body mass, for all the subordinate males both traits increase with body mass (Additional file 1: Figure S1).

**Table 1:** Summary from the linear mixed models exploring the role of social rank explaining variation in sperm design and total sperm length before manipulating the social status

	Sperm design			Total sperm length		
Fixed effects	Slope ± SD	F (*df*1, *df*2)	*p*	Slope ± SD	F (*df*1, *df*2)	*p*
Intercept	−0.79 ± 0.86			102.28 ± 0.79		
Rank		1.72 (3,34)	0.18		0.88 (3,33.9)	0.46
Subordinate 1	1.7 ± 1.23			1.35 ± 1.12		
Subordinate 2	1.43 ± 1.21			1.09 ± 1.1		
Subordinate 3	−0.66 ± 1.21			−0.08 ± 1.1		
Centred body mass	0.99 ± 0.86	4.00 (1,44.6)	*0.052*	0.77 ± 0.79	4.82 (1,43.9)	**0.033**
Centred tarsus length	1.15 ± 1.71	1.10 (1,46.4)	0.30	−0.14 ± 1.57	2.70 (1,46.8)	0.11
Rank x Centred body mass		1.70 (3,46.4)	0.18		1.25 (3,46.6)	0.30
Subordinate 1	0.65 ± 1.2			0.33 ± 1.1		
Subordinate 2	−1.76 ± 1.16			− 1.14 ± 1.07		
Subordinate 3	0.31 ± 1.09			0.75 ± 1.01		
Rank x Centred tarsus length		0.78 (3,45.5)	0.51		0.46 (3,45.5)	0.71
Subordinate 1	−3.96 ± 2.52			−2.11 ± 2.31		
Subordinate 2	−2.07 ± 2.03			−0.47 ± 1.86		
Subordinate 3	−1.83 ± 2.21			− 1.82 ± 2.04		

Estimates from linear mixed models, and F and *p* values correspond to an ANOVA using a Kenward-Roger approximation to the degrees of freedom. Contrasts are done against the means of dominant males. Bold *p*-values are significant (alpha = 0.05)

**Table 2:** Summary from the linear mixed models exploring the role of social rank explaining variation in sperm design and total sperm length after manipulating the social status

	Sperm design			Total sperm length		
Fixed effects	Slope ± SD	F (*df*1, *df*2)	*p*	Slope ± SD	F (*df*1, *df*2)	*p*
Intercept	0.33 ± 0.79			102.18 ± 0.73		
Rank		0.80 (3,33.4)	0.50		1.01 (3,33.4)	0.40
Subordinate 1	−0.77 ± 1.11			−0.24 ± 1.04		
Subordinate 2	−0.58 ± 1.09			−0.48 ± 1.02		
Subordinate 3	0.77 ± 1.09			1.14 ± 1.02		
Centred body mass	−0.59 ± 0.51	1.68 (1,44.3)	0.20	−0.49 ± 0.47	2.96 (1,44.3)	0.09
Centred tarsus length	1.28 ± 1.11	2.85 (1,42.6)	0.10	1.99 ± 1.04	1.74 (1,42.6)	0.19
Rank x Centred body mass		2.86 (3,42.9)	**0.048**		2.88 (3,42.9)	**0.047**
Subordinate 1	1.11 ± 1.08			0.75 ± 1.01		
Subordinate 2	2.67 ± 0.9			2.26 ± 0.84		
Subordinate 3	1.27 ± 0.76			1.64 ± 0.71		
Rank x Centred tarsus length		2.82 (3,43.2)	*0.05*		3.57 (3,43.2)	**0.022**
Subordinate 1	−1.99 ± 1.62			−3.09 ± 1.51		
Subordinate 2	−5.23 ± 2.14			−4.47 ± 1.99		
Subordinate 3	− 4.12 ± 1.59			− 4.83 ± 1.49		

Estimates from linear mixed models, and F and *p* values correspond to an ANOVA using a Kenward-Roger approximation to the degrees of freedom. Contrasts are done against the means of dominant males. Bold *p*-values are significant (alpha = 0.05)

### Relationships between sperm design and sperm function

**Fig. 1:**
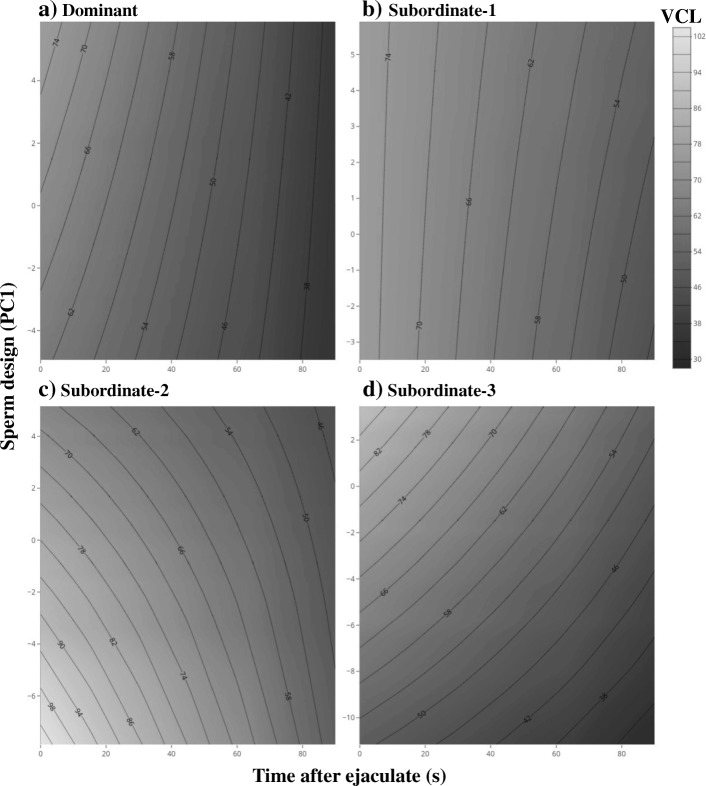
Relation between the decay in sperm velocity (VCL) through time and sperm design across social ranks (**a**-**d**) before manipulating the social environment. The surfaces were obtained from predicted values extracted from linear mixed models. Larger PC1 values indicate larger flagella and mid-pieces relative to head size, and they are centered by social rank

**Fig. 2:**
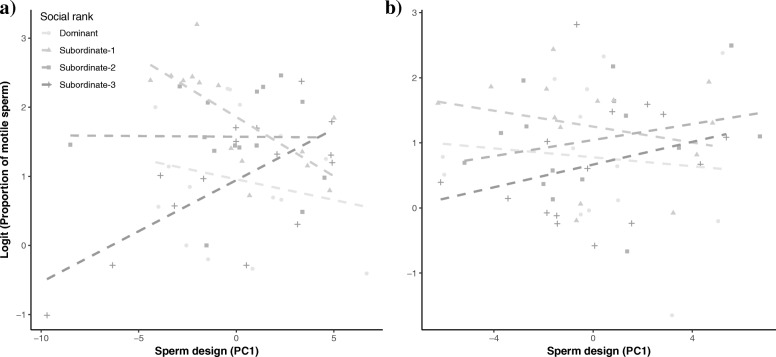
Relation between sperm design and the initial proportion of motile sperm across social ranks both (**a**) before and (**b**) after the social status manipulation. For more details, refer to Table 3

Before manipulating the social status, we found that males occupying different social status differed in their sperm velocity and proportion of motile sperm (Tables 3 and 4). Further, we found that the relation between sperm velocity and sperm design across time (e.g. ability to maintain their speed) differed across social ranks (Table 3; Fig. 1). A similar result was found for the relation between sperm velocity and total sperm length for different social ranks (Table 4; Additional file 1: Figure S2). We also found that the rate at which the proportion of motile sperm decreased with time differed between social ranks (Tables 3 and 4). Finally, our results revealed that the relation between sperm design and the initial proportion of motile sperm also differed between social ranks (Table 3; Fig. 2A).

**Table 3:** Relation between sperm functional traits and sperm design

	Before social status manipulation						After social status manipulation					
Fixed effects	VCL			Proportion of motile sperm			VCL			Proportion of motile sperm		
	Slope ± SD	F (*df*1,*df*2)	*p*	Slope ± SD	F (*df*1,*df*2)	*p*	Slope ± SD	F (*df*1,*df*2)	*p*	Slope ± SD	F (*df*1,*df*2)	*p*
Intercept	−29.64 ± 72.88			−4.79 ± 4.24			79.4 ± 73.83			−3.92 ± 4.28		
Social rank		3.13 (3,36)	**0.037**		5.62 (3,36.1)	**0.003**		1.08 (3,34.9)	0.37		0.89 (3,35.5)	0.45
Subordinate 1	9.91 ± 5.56			0.94 ± 0.32			9.43 ± 6.15			0.59 ± 0.37		
Subordinate 2	14.94 ± 5.84			0.89 ± 0.34			−0.48 ± 6.12			0.32 ± 0.36		
Subordinate 3	8.96 ± 5.46			0.14 ± 0.32			2.03 ± 6.13			0.08 ± 0.37		
Sperm design	0.81 ± 1.33	0.7 (1,42.2)	0.41	−0.07 ± 0.08	2.87 (1,42.9)	0.1	0.37 ± 1.46	0.40 (1,47.9)	0.53	−0.08 ± 0.08	0.001 (1,44.5)	0.99
Time	−0.37 ± 0.03	563.62 (1287)	**> 0.001**	− 0.02 ± 0.001	367.22 (1287)	**> 0.001**	−0.36 ± 0.03	617.62 (1282)	**> 0.001**	−0.009 ± 0.001	238.64 (1282)	**< 0.001**
Body mass	2.86 ± 1.83	2.36 (1,43.4)	0.13	0.09 ± 0.11	0.76 (1,44.2)	0.39	2.59 ± 1.83	1.92 (1,43.2)	0.17	0.01 ± 0.11	0.01 (1,46.9)	0.92
Tarsus length	0.98 ± 3.23	0.09 (1,45.5)	0.77	0.16 ± 0.19	0.66 (1,46.2)	0.42	−3.85 ± 3.63	1.10 (1,38)	0.30	0.22 ± 0.21	1.05 (1,41.1)	0.31
Social rank x Sperm design		1.06 (3,46.1)	0.37		3.09 (3,46.7)	**0.036**		1.03 (3,43)	0.39		1.46 (3,46)	0.24
Subordinate 1	−1.24 ± 1.97			−0.05 ± 0.11			−2.11 ± 2.03			0.02 ± 0.12		
Subordinate 2	−2.9 ± 1.92			0.12 ± 0.11			1.59 ± 2.02			0.17 ± 0.12		
Subordinate 3	1.5 ± 1.68			0.24 ± 0.10			−1.6 ± 2.28			0.20 ± 0.13		
Social rank x Time		0.99 (3287)	0.4		8.83 (3287)	**> 0.001**		1.34 (3282)	0.26		1.73 (3282)	0.16
Subordinate 1	0.06 ± 0.04			0.005 ± 0.002			0.01 ± 0.04			−0.002 ± 0.002		
Subordinate 2	0.001 ± 0.04			0.005 ± 0.002			0.06 ± 0.04			−0.004 ± 0.002		
Subordinate 3	0.02 ± 0.04			0.01 ± 0.002			0.06 ± 0.04			−6.0 E-4 ± 0.002		
Sperm design x Time	−0.01 ± 0.01	0.06 (1287)	0.81		1.83 (1287)	0.18	0 ± 0.01	5.78 (1282)	**0.017**	−2.7 E-4 ± 4.3 E-4	0.56 (1282)	0.45
Social rank x Sperm design x Time		4.34 (3287)	**0.005**	8.2 E-5 ± 4.8 E-4	2.4 (3287)	*0.07*		1.74 (3282)	0.16		1.55 (3282)	0.20
Subordinate 1	0.02 ± 0.01			0.001 ± 7.1 E-4			−0.01 ± 0.01			−2.5 E-5 ± 6.2 E-4		
Subordinate 2	0.04 ± 0.01			−3.5 E-5 ± 6.8 E-4			−0.02 ± 0.01			7.9 E-4 ± 6.0 E-4		
Subordinate 3	0.001 ± 0.01			7.1 E-5 ± 6.0 E-4			0 ± 0.01			−5.1 E-4 ± 6.4 E-4		

Estimates from linear mixed models, and F and *p* values from ANOVAs with Kenward-Roger approxi-mation to the degrees of freedom. Contrast are done against the dominant males. Bold *p*-values are significant (alpha = 0.05)

**Table 4:** Relation between sperm functional traits and total sperm length

	Before social status manipulation						After social status manipulation					
Fixed effects	VCL			Proportion of motile sperm			VCL			Proportion of motile sperm		
	Slope ± SD	F (*df*1,*df*2)	*p*	Slope ± SD	F (*df*1,*df*2)	*p*	Slope ± SD	F (*df*1,*df*2)	*p*	Slope ± SD	F (*df*1,*df*2)	*p*
Intercept	−43.87 ± 71.08			−6.05 ± 4.18			75.36 ± 74.99			−3.86 ± 4.28		
Social rank		3.04 (3,36.1)	**0.041**		5.51 (3,36.1)	**0.003**		1.04 (3,34.9)	0.39		0.87 (3,35.5)	0.46
Subordinate 1	10.06 ± 5.61			0.97 ± 0.33			9.73 ± 6.29			0.59 ± 0.37		
Subordinate 2	15.42 ± 5.87			0.94 ± 0.34			−0.42 ± 6.26			0.32 ± 0.37		
Subordinate 3	9.15 ± 5.51			0.17 ± 0.32			2.14 ± 6.27			0.07 ± 0.37		
Sperm total length	1.64 ± 1.45	0.0003 (1,47.1)	0.98	−0.11 ± 0.08	0.68 (1,47.2)	0.41	0.63 ± 1.43	0.1 (1,47.7)	0.76	−0.08 ± 0.08	0.001 (1,46.3)	0.99
Time	−0.37 ± 0.03	561.43 (1287)	**> 0.001**	− 0.02 ± 0	357.45 (1287)	**> 0.001**	−0.36 ± 0.03	617.31 (1282)	**> 0.001**	−0.01 ± 0	235.71 (1282)	**> 0.001**
Body mass	3.19 ± 1.79	3.05 (1,42.8)	0.09	0.09 ± 0.11	0.75 (1,42.9)	0.39	2.95 ± 1.88	2.36 (1,42.8)	0.13	0.01 ± 0.11	0.01 (1,45.9)	0.91
Tarsus length	1.24 ± 3.23	0.14 (1,46.3)	0.71	0.22 ± 0.19	1.31 (1,46.4)	0.26	−4.16 ± 3.74	1.21 (1,38.6)	0.28	0.22 ± 0.22	0.97 (1,40.9)	0.33
Social rank x Sperm total length		1.47 (3,44.9)	0.23		3.25 (3,45)	0.030		0.7 (3,44)	0.55		1.16 (3,46.4)	0.34
Subordinate 1	−3.26 ± 2.06			0.04 ± 0.12			−3.03 ± 2.49			0.02 ± 0.14		
Subordinate 2	−4.37 ± 2.01			0.17 ± 0.12			0.43 ± 2.07			0.12 ± 0.12		
Subordinate 3	0.2 ± 1.88			0.28 ± 0.11			−0.9 ± 2.23			0.19 ± 0.13		
Social rank x Time		0.98 (3287)	0.40		8.59 (3287)	**> 0.001**		1.34 (3282)	0.26		1.71 (3282)	0.17
Subordinate 1	0.06 ± 0.04			0.005 ± 0.002			0.01 ± 0.04			−0.002 ± 0.002		
Subordinate 2	0 ± 0.04			0.01 ± 0			0.06 ± 0.04			−0.004 ± 0.002		
Subordinate 3	0.02 ± 0.04			0.01 ± 0			0.06 ± 0.04			−0.001 ± 0.002		
Sperm design x Time	−0.02 ± 0.01	0.02 (1287)	0.89	− 0.0003 ± 0.0005	0.02 (1287)	0.89	0 ± 0.01	3.78 (1282)	*0.053*	−0.0004 ± 0.0004	0.20 (1282)	0.65
Social rank x Total length x Time		3.96 (3287)	**0.009**		0.38 (3287)	0.76		2.35 (3282)	0.07		0.49 (3282)	0.69
Subordinate 1	0.02 ± 0.01			0.0007 ± 0.0008			−0.01 ± 0.01			−0.0001 ± 0.0007		
Subordinate 2	0.04 ± 0.01			0.0001 ± 0.0007			−0.03 ± 0.01			0.0006 ± 0.0006		
Subordinate 3	0.01 ± 0.01			0.0005 ± 0.0007			−0.01 ± 0.01			0.0003 ± 0.0006		

Estimates from linear mixed models, and F and *p* values from ANOVAs with Kenward-Roger approximation to the degrees of freedom. Contrast are done against the dominant males. Bold *p*-values are significant (alpha = 0.05)

**Fig. 3:**
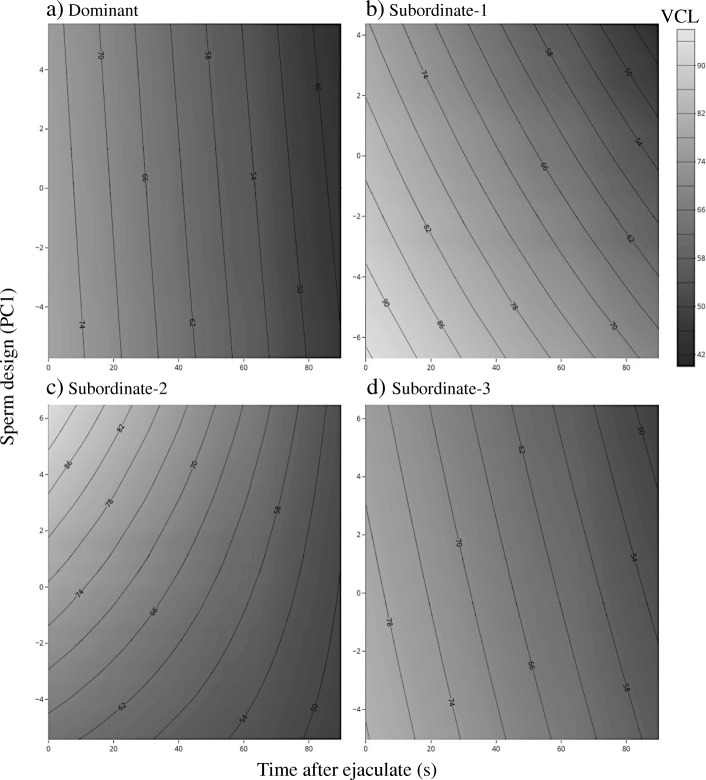
Relation between the decay in sperm velocity (VCL) across time and sperm design across social ranks (**a**-**d**) after manipulating the social environment. The surfaces were obtained from predicted values extracted from linear mixed models. Larger PC values indicate larger flagella and mid-pieces relative to head size, and they are centered by social rank

After manipulating the social status, we found no differences across ranks for the relationship between sperm velocity and sperm design (Table 3; Fig. 3) or total sperm length (Table 4; Additional file 1: Figure S3). Further, the relation between sperm design and the initial proportion of motile sperm disappeared after the social status manipulation (Table 3; Fig. 2B)

## Discussion

In this study, we aimed at testing whether sperm performance is functionally related to sperm morphology in a passerine bird, the house sparrow. Based on results from a previous study showing that sperm performance varies according to male social status, we predicted that, if sperm morphology explains variation in sperm performance, 1) sperm morphology should differ across social ranks, and 2) the direction of the correlations between sperm morphology and sperm velocity and/or sperm longevity should remain similar across all social ranks. We found no differences in sperm morphology across social ranks (Table 1). Moreover, we found that the correlations between sperm velocity and sperm design (Fig. 1) or total sperm length (Additional file 1: Figure S2) depended on male social rank. Specifically, we found that among dominant and subordinate-1 male sperm design (the size of the flagellum and the midpiece relative to the size of the sperm head) was unrelated to the rate at which sperm velocity decays through time. In contrast, subordinate-2 males producing spermatozoa with shorter flagellum and mid-piece relative to the head (Fig. 1) suffered from a steeper decay in sperm velocity through time. Finally, the pattern was opposite in subordinate-3 males. Similar patterns were observed for total sperm length (Additional file 1: Figure S2). Therefore, our results do not support the hypothesis that sperm morphology is functionally related to sperm swimming ability, and sperm morphology cannot explain the covariation between sperm motility and social status observed previously [49].

It has been previously hypothesized that larger flagella and mid-pieces help spermatozoa generate more thrust while maintaining high energetic demands that are required to sustain a larger flagellum, which in turn could lead to a faster swimming speed [29]. Our results provide, at best, mixed support for this hypothesis (see Fig. 1). Moreover, after experimentally manipulating the social environment we did not observe changes in either sperm design or total sperm length that matched the new social ranks, and most of the previously observed correlations between sperm design and swimming performance disappeared or changed direction (Fig. 2B, 3, and Additional file 1: Figure S3). These results further challenge the idea of a functional relationship between sperm morphology and sperm performance (e.g. velocity, longevity, motility), and rather suggest that many of the observed correlations do not reflect causality between the two traits.

In House Sparrows, a previous study found that sperm with bigger heads relative to the flagellum swim at a lower speed [33]. Yet, another study found the opposite relationship [50]. Here, we found that the relationships between sperm design and sperm performance varied depending on male social status. Thus, it could be argued that sampling bias towards bolder, more dominant vs. shier, more subordinate males might explain the discrepancies between the two previous studies. After we experimentally manipulated the social environment, many of the observed correlations between sperm morphology and performance disappeared. However, our previous data on house sparrows suggest that males are able to adjust their sperm quality to match their new social ranks [49], and the results of the present study indicate that such adjustments do not require any changes in sperm morphology.

Previous comparative studies have found differences in sperm morphology across species characterized by different risks of sperm competition [15, 17, 51, 52]. In house sparrows, large between-male variation in sperm morphology has been reported [33]. Yet, neither sperm performance nor social dominance explain why males differ so much in sperm morphology (this study). Plasticity in sperm morphology has been also reported in passerine birds, but whether such changes in morphology would result in changes in sperm performance is not clear. Specifically, it has been found that an experimental manipulation of the perceived male-male competition levels leads to changes in sperm morphology in Gouldian Finches [53]. Here, we did not find any changes in sperm morphology after experimentally modifying males’ social status, which is expected given the lack of correlation between sperm performance and morphology.

A possible explanation for why sperm performance covaries with social rank [49] and sperm morphology does not (the current study) is that sperm morphology is constrained by the morphology of the female sperm storage organs. Between bird species, a positive correlation between avian sperm size and the length of the sperm storage tubules (SSTs) suggests that the female reproductive tract can exert selection on sperm size [54]. Thus within species, stabilizing selection towards an optimal sperm length that matches SSTs could constrain plasticity in sperm morphology, while post-copulatory sexual selection (either through sperm competition or female cryptic choice) may still favor other mechanisms that enhance ejaculate fertilizing ability to match the risk of sperm competition faced by a male. Consequently, we suggest that these two different selection forces may eventually decoupled sperm morphology from sperm performance in the case of house sparrows.

It has been found that increasing levels of sperm competition might select for larger sperm mitochondrial loads in primates [11], while evidence in other taxa has shown that sperm ATP content is positively correlated with sperm motility [55–57]. For instance, males at different social ranks may differ in the initial ATP stores in their sperm cells e.g. ATP; [58, 59], and thus males might be able to adjust their sperm motility without changing their sperm morphology. Yet, mitochondrial energy production can release by-products that can cause oxidative damage [60], which disrupts sperm membranes [61]. Indeed, it has been observed that sperm swimming ability is negatively correlated with the levels of membrane oxidation [62, 63]. Consequently, rather than modifying their sperm morphology, males may be selected to modify their sperm performance via differential resource allocation to protect sperm from ROS, a hypothesis supported by results showing that the levels of oxidative damage to sperm vary across social ranks [49]. Alternatively, seminal fluid composition has been shown to have a positive impact on sperm performance [64], and indeed it has been observed in fowls that dominant males can strategically inseminate females with faster swimming sperm due to the seminal fluids used for the ejaculate [65].

## Conclusion

To conclude, we found that, in house sparrows, sperm morphology does not reflect sperm performance, yet it is worth noting that our assays were done in an artificial medium that does not necessarily reflect the conditions experience by sperm within the female reproductive tract. Nonetheless, we believe that while sperm morphology might be constrained by the length of the female sperm storage tubules, selection may still act upon sperm physiology to enhance traits associated with sperm performance. We suggest that, as a consequence of such different selective pressures, sperm design may be functionally decoupled from sperm performance. Finally, we encourage researchers focusing on the relationships between sperm morphology and sperm performance to include both information about the males’ reproductive opportunities (e.g. risk of sperm competition) and sperm physiology.

## Methods

### Individuals and sampling

We trapped 60 male and 60 female House Sparrows in western Switzerland in April 2014. Before transferring birds to 15 mixed outdoor aviaries at the Hasli Ethological Station (University of Bern, Switzerland), we measured their body mass and tarsus length. Additionally, birds were scored for their badge size (1–5 following the diagram in Fig. 1 from [66]) and assigned to their aviaries based both on their badge size score and their body mass to ensure that all aviaries would contain approximately the same distribution of small-badged, medium-badge and large-badged males and thus would exhibit approximately the same distribution in relative badge size (and potentially competitive ability), and to ensure that average body mass did not significantly differ across aviaries. Males in each aviary were banded with either a red, white, yellow, or orange color ring and a unique ID metal ring. After four weeks of acclimatization, females were transferred to a separate aviary, and we took a sperm sample from each male. We then collected a second sperm sample the day after, and a third sperm sample after 48 h from the last sample. This procedure ensured that any differences in sperm characteristics would be intrinsic differences in quality rather than differences due to depletion [40, 67] or fresh sperm effects [68–70]. To maintain sampling time within reasonable limits per day, males were divided in three sampling batches of five aviaries, and batches were processed with a 5-day gap.

Females were then reintroduced to the aviaries and males were shuffled between aviaries according to their initial social rank, as to optimize the number of movements upwards and downwards in the hierarchies (For details on the procedure, see Additional file 2: Table S1). Males were given three weeks to settle into their new social ranks, to ensure that all males went through at least one spermatogenic cycle [71]. Finally, a sperm sampling session was done as described above.

### Behavioral observations and assessment of social ranks

During the acclimatization period, we performed a total of 13 h of video recordings on each aviary to establish the hierarchical position of each male, and recordings were done once a day for one hour. Each aviary had a tower feeder mounted on a plastic plate that collected all the spilt seeds through a plastic mesh, making food only accessible at the two feeder openings. Feeders were removed each morning for 90 min, and a GoPro camera was introduced with the feeders. From the videos, we counted the number of antagonistic interactions (fights and chasing) between males, and used those data to calculate a David’s score [72]. After manipulating the social status, we established the social ranks from 10 h of video from each aviary, following the same procedures.

### Sperm morphology and sperm swimming performance

Samples were obtained by gently massaging males’ cloacae [73], and ejaculates were collected in 5 μl glass capillaries. Right after collection, 0.25 μL of ejaculate were diluted in 40 μL of preheated Dulbecco Modified Eagle Medium at 40 °C, and then a 20 μm chamber (Leja Products B.V., The Netherlands) was loaded with the diluted ejaculate. Immediately after loading the chamber, a video was done using a Toshiba CMOS HD camera (TOSHIBA Corporation, Japan) camera mounted on a light microscope at 100× magnification and phase contrast 3 annular ring, while keeping a constant temperature of 40 °C with a heating plate mounted on the microscope (Minitube HT200 W, MINITÜB GmbH, Germany). A small droplet from the ejaculate was smeared with 10% formalin on a glass slide for sperm morphology assessments. From each sample, we took photos of ten sperm cells, with coiled midpiece and unbroken heads, using the Nikon ACT-1 v2.70 software (Nikon Corporation, Japan) for a Nikon Digital Eclipse DXM1200 camera (Nikon Corporation, Japan) mounted on a Leica DM R microscope (Leica Microsystems GmbH, Germany) at 400× magnification and phase contrast 2.

From the videos, we estimated the curvilinear velocity (VCL) and the proportion of motile sperm using a computer automated sperm analyser plug-in [74] for ImageJ [75]. Sperm cells having a VSL < 5 μm/s, a VCL < 15 μm/s, or a VAP < 10 μm/s were considered as either moved by drift or immotile. Each video was sampled at 0, 15, 30, 60 and 90 s after the beginning of the recording, thus allowing us to estimate sperm longevity and sperm ability to maintain initial speed. From each photo, we measured the straight head, midpiece, flagellum, and total length, and S.C. made all the morphological measurements to avoid errors due to observer bias in the measurements. Each photo was independently measured twice blind to the previous measurements, and the average between the two measurements was used for further analyses. All samples were processed blind to male identity and social rank.

### Statistical analyses

For each male, we calculated the average in sperm morphology from the 10 spermatozoa, and then we used these mean measures in further analyses. To estimate sperm design, we performed a principal component analysis using head, mid-piece, and flagellum lengths, and we then extracted the first component as a measurement of sperm design (Rotations PC1: head length = 0.008, mid-piece length = 0.59, flagellum length = 0.81; Proportion of variance explained by PC1 = 0.74). Thus, males with higher scores along the PC1 produced spermatozoa with longer mid-pieces and flagella, relative to sperm head size. To test whether males at different social ranks differed in sperm morphology or design, we ran linear mixed models using total sperm length or sperm design as response variable and social rank as a predictor variable. Additionally, body weight and tarsus length, both centered on rank, were used as covariates as well as their interactions with social rank, while the aviary and the collection batch were entered as random intercepts.

To test whether total sperm length or sperm design would explain variation in sperm swimming performance, we performed linear mixed models using the proportion of motile sperm and the curvilinear velocity (VCL) as response variables. The predictor variables were the social rank, total sperm length or sperm design centered on rank, the time after the video recording started, and the interactions between them, while body weight and tarsus length were used as covariates. The models included the aviary and male identity as random intercepts, as well as the interaction between male identity and time to model random slopes.

After experimentally modifying the social environments, we tested whether the patterns observed before manipulating the social status were maintained. Thus, we ran similar models but using the data collected after the social status manipulation, as well as the newly stablished social ranks.

In all models, proportion of motile sperm was logit transformed to match normality. All the models were done using a restricted maximum likelihood method for parameter estimation, and Kenward-Roger approximation of fixed effects degrees of freedom. We did not apply model selection to avoid inflating the type I error probability [76]. All the analyses were performed using R v 3.3.2.

## Additional files

Additional file 1: **Figure S1.** Relation between sperm morphological design and male body mass centered by social ranks. Lines represent a linear regression, whereas colors indicate dominant (D) and subordinate- 1 to 3 (S1–3) males. **Figure S2.** Relation between the decay in sperm velocity (VCL) through time and sperm total length across social ranks before manipulating the social environment. The surfaces were obtained from predicted values extracted from linear mixed models. Values are centered by social rank. **Figure S3.** Relation between the decay in sperm velocity (VCL) through time and sperm total length across social ranks after manipulating the social environment. The surfaces were obtained from predicted values extracted from linear mixed models. Values are centered by social rank. (DOCX 2323 kb)

Additional file 2: **Table S1.** Male social status manipulation. The manipulation was performed equally for each experimental block of 5 aviaries, and the net change reflects the number of social ranks that individual were expected to gain or lose (the net change adds up to zero). (XLSX 9 kb)
