# Supplementary material for: Is sperm morphology functionally related to sperm swimming ability? A case study in a wild passerine bird with male hierarchies
Source: BMC Evol Biol. 2018 Sep 19;18:142. doi: 10.1186/s12862-018-1260-8 (PMC6146611; doi:10.1186/s12862-018-1260-8)
Supplement: Supplementary file 1 — Figure S1. Relation between sperm morphological design and male body mass centered by social ranks. Lines represent a linear regression, whereas colors indicate dominant (D) and subordinate- 1 to 3 (S1–3) males. Figure S2. Relation between the decay in sperm velocity (VCL) through time and sperm total length across social ranks before manipulating the social environment. The surfaces were obtained from predicted values extracted from linear mixed models. Values are centered by social rank. Figure S3. Relation between the decay in sperm velocity (VCL) through time and sperm total length across social ranks after manipulating the social environment. The surfaces were obtained from predicted values extracted from linear mixed models. Values are centered by social rank. (DOCX 2323 kb) [file 12862_2018_1260_MOESM1_ESM.docx]

**Additional file 1**

**Is sperm morphology functionally related to sperm swimming ability? A case study in a wild passerine bird with male hierarchies**

Alfonso Rojas Mora^1,*^, Magali Meniri^1^, Sabrina Ciprietti^1^, Fabrice Helfenstein^1,*^

^1^Laboratory of Evolutionary Ecophysiology, Institute of Biology, Faculty of Sciences, University of Neuchatel, Neuchatel, Switzerland

^*^Correspondence to: FH. Rue Emile-Argand 11, Institute of Biology, Faculty of Sciences, University of Neuchatel, Neuchatel, Switzerland.
Phone: +41 32 718 2234. E-mail: [fabrice.helfenstein@unine.ch](mailto:fabrice.helfenstein@unine.ch)

^*^Correspondence to: ARM. Rue Emile-Argand 11, Institute of Biology, Faculty of Sciences, University of Neuchatel, Neuchatel, Switzerland.
Phone: +41 32 718 3037. E-mail

**Figure S1.** Relation between sperm morphological design and male body mass centered by social ranks. Lines represent a linear regression, whereas colors indicate dominant (D) and subordinate- 1 to 3 (S1-3) males.

**Figure S2.** Relation between the decay in sperm velocity (VCL) through time and sperm total length across social ranks before manipulating the social environment. The surfaces were obtained from predicted values extracted from linear mixed models. Values are centered by social rank.

**Figure S3.** Relation between the decay in sperm velocity (VCL) through time and sperm total length across social ranks after manipulating the social environment. The surfaces were obtained from predicted values extracted from linear mixed models. Values are centered by social rank.
